# A Senescence Bystander Effect in Human Lung Fibroblasts

**DOI:** 10.3390/biomedicines9091162

**Published:** 2021-09-04

**Authors:** David W. Waters, Michael Schuliga, Prabuddha S. Pathinayake, Lan Wei, Hui-Ying Tan, Kaj E. C. Blokland, Jade Jaffar, Glen P. Westall, Janette K. Burgess, Cecilia M. Prêle, Steven E. Mutsaers, Christopher L. Grainge, Darryl A. Knight

**Affiliations:** 1 School of Biomedical Sciences and Pharmacy, University of Newcastle, Callaghan, NSW 2308, Australia; David.W.Waters@uon.edu.au (D.W.W.); lan.wei@uon.edu.au (L.W.); c3348759@uon.edu.au (H.-Y.T.); k.e.c.blokland@umcg.nl (K.E.C.B.); Darryl.Knight@hli.ubc.ca (D.A.K.); 2 National Health and Medical Research Council Centre of Research Excellence in Pulmonary Fibrosis, New Lambton Heights, NSW 2308, Australia; 3 School of Medicine and Public Health, University of Newcastle, Callaghan, NSW 2308, Australia; Prabuddha.Pathinayake@uon.edu.au (P.S.P.); christopher.grainge@hnehealth.nsw.gov.au (C.L.G.); 4 University Medical Center Groningen, Groningen Research Institute for Asthma and COPD (GRIAC), University of Groningen, 9713 GZ Groningen, The Netherlands; j.k.burgess@umcg.nl; 5 Allergy, Immunology and Respiratory Medicine, Alfred Hospital, Melbourne, VIC 3004, Australia; jade.jaffar@monash.edu (J.J.); G.Westall@alfred.org.au (G.P.W.); 6 Centre for Cell Therapy and Regenerative Medicine, School of Biomedical Sciences, University of Western Australia, Nedlands, WA 6009, Australia; cecilia.prele@uwa.edu.au (C.M.P.); steven.mutsaers@uwa.edu.au (S.E.M.); 7 Institute for Respiratory Health, University of Western Australia, Nedlands, WA 6009, Australia; 8 Providence Health Care Research Institute, Vancouver, BC V6Z 1Y5, Canada

**Keywords:** collagen, idiopathic pulmonary fibrosis (IPF), lung fibroblasts, senescence

## Abstract

Idiopathic pulmonary fibrosis (IPF) is a chronic disease characterised by a dense fibrosing of the lung parenchyma. An association between IPF and cellular senescence is well established and several studies now describe a higher abundance of senescent fibroblasts and epithelial cells in the lungs of IPF patients compared with age-matched controls. The cause of this abnormal accumulation of senescent cells is unknown but evidence suggests that, once established, senescence can be transferred from senescent to non-senescent cells. In this study, we investigated whether senescent human lung fibroblasts (LFs) and alveolar epithelial cells (AECs) could induce a senescent-like phenotype in “naïve” non-senescent LFs in vitro. Primary cultures of LFs from adult control donors (Ctrl-LFs) with a low baseline of senescence were exposed to conditioned medium (CM) from: (i) Ctrl-LFs induced to become senescent using H_2_O_2_ or etoposide; (ii) LFs derived from IPF patients (IPF-LFs) with a high baseline of senescence; or (iii) senescence-induced A549 cells, an AEC line. Additionally, ratios of non-senescent Ctrl-LFs and senescence-induced Ctrl-LFs (100:0, 0:100, 50:50, 90:10, 99:1) were co-cultured and their effect on induction of senescence measured. We demonstrated that exposure of naïve non-senescent Ctrl-LFs to CM from senescence-induced Ctrl-LFs and AECs and IPF-LFs increased the markers of senescence including nuclear localisation of phosphorylated-H2A histone family member X (H2AXγ) and expression of p21, IL-6 and IL-8 in Ctrl-LFs. Additionally, co-cultures of non-senescent and senescence-induced Ctrl-LFs induced a senescent-like phenotype in the non-senescent cells. These data suggest that the phenomenon of “senescence-induced senescence” can occur in vitro in primary cultures of human LFs, and provides a possible explanation for the abnormal abundance of senescent cells in the lungs of IPF patients.

## 1. Introduction

Senescent cells are a key yet transient feature of tissue homeostasis contributing to processes such as wound repair and resolution [1]. Typically, senescent cells are eliminated from the environment by infiltrating immune cells to maintain tissue homeostasis. However, when clearance of these cells becomes compromised, for example, due to an ageing immune system, they accumulate in tissues [2]. By their nature, senescent cells secrete a variety of bioactive molecules including chemokines, growth factors and reactive oxygen species (ROS) that engage in autocrine and paracrine interactions to influence the local microenvironment [3,4,5]. Senescence can result from excessive mitotic division, also referred to as mitotic ageing, or as a response to severe stress. The origin of the stressing stimuli in vivo has been difficult to identify but it is now apparent that senescence can be propagated through a cell population from senescent to non-senescent cells in a non-autonomous manner. This spread of senescence has been termed “senescence-induced senescence” [5].

Idiopathic pulmonary fibrosis (IPF), a chronic lung disease that occurs primarily in the elderly, is characterised by the accumulation of fibroblasts and collagen in the lung parenchyma [6,7]. As the disease progresses, the normal architecture of the lung is remodelled through deposition of excessive amounts of extracellular matrix (primarily collagen), which irreversibly inhibits oxygen transfer. This results in significant morbidity and mortality, with survival of 3–5 years following diagnosis [8]. A number of studies have reported a higher abundance of senescent fibroblasts and epithelial cells in the lungs of IPF patients compared to age-matched controls [9,10]. Senescent lung fibroblasts (LFs) are likely to play a role in IPF pathogenesis, exhibiting myofibroblast-like characteristics (i.e., increased α-smooth muscle actin and collagen Iα1 expression), apoptosis resistance and a highly activated secretome [1,9,10,11,12,13,14,15,16]. A failure to eliminate senescent fibroblasts by apoptosis or immune cell clearance is thought to contribute to the aberrant wound repair response that underlies disease progression in IPF [9,11].

In this study we explored the transfer of senescence (the “bystander effect”) between LFs and alveolar epithelial cells (AECs), a phenomenon that may contribute to the accumulation of senescent cells in the IPF lung. These investigations are unique in that they use primary cultures of human LFs from age-appropriate adult donors, including patients with IPF. In particular, we describe a novel co-culture model that does not rely on reporter cell lines, as has been used previously in comparable studies [5]. We first examined the effect of culturing non-senescent control (Ctrl)-LFs with conditioned medium (CM) from senescent-induced Ctrl-LFs and AECs, as well as IPF-LFs with high baseline senescence. We then used a co-culture system whereby senescence-induced Ctrl-LFs labelled with a fluorescent dye were seeded and grown with non-labelled naïve Ctrl-LFs. Our data showed that senescent LFs and AECs were able to induce a senescent-like phenotype in “bystander” LFs in vitro. This suggests that senescent lung cells secrete senescence-inducing soluble bioactive mediators.

## 2. Materials and Methods

### 2.1. Lung Tissue

Lung tissue from IPF patients was obtained from the Alfred Lung Fibrosis Biobank (Alfred Hospital, Melbourne, Australia) under ethical approval from the Alfred Health Ethics Committee (#336/13) following National Health and Medical Research Council (NHMRC, Australia) guidelines. Primary cultures of lung fibroblasts were established from macroscopically normal parenchymal tissue of lung donors at the Alfred hospital (Melbourne, Victoria, Australia) or patients with non-fibrosing lung diseases (i.e., cancer) undergoing thoracic surgery at the John Hunter Hospital (Newcastle, NSW, Australia). These non-IPF cultures of lung fibroblasts were referred to as controls (Ctrl). Tissue was obtained with informed written consent under ethical approval from the human research ethics committees of the hospitals (#336/13, approved 26 November 2013 and HNEHREC 16/07/20/5.03, approved 16 July 2016) and the University of Newcastle (H-2016-0325, approved 26 October 2016) and followed NHMRC guidelines. Demographics of patients are provided in Table 1.

### 2.2. Immunohistochemistry

Serial sections of paraffin-embedded parenchymal lung tissue from IPF patients were immunolabelled for p21 using a rabbit polyclonal antibody (Cell Signaling Technology, Danvers, MA, USA, #2947, diluted 1 in 50) and detected using the Dako EnVision anti-rabbit kit (Dako Corp., Carpinteria, CA, USA) and 3,3′-diaminobenzidine (DAB, Sigma-Aldrich, St Louis, MO, USA); or stained with Masson’s Trichrome to identify collagen. Masson’s trichrome staining was performed at The Hunter Medical Research Institute’s (HMRI’s) Pathology Service laboratory and 40× magnification images digitalised using the Aperio AT2 digital scanner (Leica, Wetzlar, Hesse, Germany).

### 2.3. Dual Immunofluorescence Detection of p21 and α-SMA in Lung Tissue

Lung sections were co-stained for p21 and α-SMA by immunofluorescence. Antigens were identified by rabbit polyclonal antibodies to p21 (Cell Signaling Technology #2947, diluted 1 in 50) and monoclonal mouse antibodies to α-SMA (#A2547, Sigma, diluted 1 in 200). Primary antibodies bound to antigen were detected using Alexa Fluor 555 anti-rabbit-conjugate (Cell Signaling Technology, Danvers, MA, USA) or Alexa Fluor 488 anti-mouse conjugate (Cell Signaling Technology). Secondary antibodies were used at a 1:1000 dilution. Tissues were mounted under coverslips using Prolong Gold AntiFade with DAPI (Molecular Probes, Cell Signaling Technology) and fluorescent images were captured at 100× magnification using a Nikon Eclipse Ti-U fluorescence microscope.

### 2.4. Cell Culture

To establish primary cultures of lung fibroblasts, lung tissue was minced into ~2 mm^3^ pieces and allowed to adhere to plastic culture plates for 10 min in the absence of medium. Tissue samples were then incubated in Dulbecco’s Modified Eagle’s Medium (DMEM) GlutaMAX Low Glucose (ThermoFisher Scientific, Scoresby, VIC, Australia) supplemented with HEPES (16 mM), 10% *v*/*v* Fetal Calf Serum (FCS), penicillin (50 U/mL), streptomycin (50 µg/mL), 2.5 µg/mL amphotericin B (all from Sigma-Aldrich) at 37 °C in air containing 5% CO_2_. Cells were allowed to migrate out from the tissue and grown to confluence over 3–4 weeks, at which point tissue pieces were removed. Adherent cells were passaged when the culture flasks were ~90% confluent. Later passages were maintained in DMEM containing 10% *v*/*v* FCS. Experiments were performed with fibroblasts between passages 2 to 6 and seeded into tissue culture plates at a density of 1.5 × 10^4^ cells per cm^2^. After 24 h, cells were replenished in serum-reduced DMEM (containing 0.4% *v*/*v* FCS) for experimentation.

### 2.5. Senescence Induction Protocol

Fibroblasts maintained in DMEM containing 0.4% *v*/*v* FCS for 24 h were then exposed to 150 µM H_2_O_2_ (Merck, Darmstadt, Hesse, Germany) for 2 h. Medium from both treated and untreated control fibroblasts were removed, the cells washed twice with PBS and then maintained in fresh DMEM containing 0.4% *v*/*v* FCS for an additional 3 d. Using this protocol, H_2_O_2_-treated cells exhibit features of senescence after 3 d, including increased levels of p21, senescence associated β-galactosidase (SA-β-gal) activity and cytokine (IL-6) production [16,17]. Fibroblast and A549 cellular senescence was also induced using etoposide. Like the H_2_O_2_-induction protocol, cells were exposed for 2 h to etoposide (10 μM for fibroblasts and 3 μM for A549 cells), before being replenished in fresh serum-reduced medium. After a period of 3 days, etoposide-treated cells also exhibited a senescence phenotype [14,15].

### 2.6. Conditioned Medium (CM) Transfer Experiments

Cultures of naïve “donor” cells (i.e., Ctrl-LFs or A549s cells) were induced into a senescence phenotype by exposure to H_2_O_2_ or etoposide for 2 h, before the medium was replenished with fresh medium without H_2_O_2_ or etoposide. Fibroblasts were then cultured for an additional 3 d before the CM was used to treat “recipient” naïve lung fibroblasts for a further 3 d. Cells were then either fixed for fluorescence microscopy or lysed for Western blot or mRNA analysis.

### 2.7. Cell Enumeration

Naïve recipient lung fibroblasts maintained in CM for 3 d in 24 well plates were replenished with fresh DMEM containing 5% *v*/*v* FCS. After 48 h, attached cells were dissociated and harvested by incubation with trypsin (0.125% *w*/*v*) and EDTA (0.02% *w*/*v*) in PBS. Cells were resuspended in 5% *v*/*v* FCS in PBS containing trypan blue (0.2% *w*/*v*) and viable cells counted (in duplicate) with the aid of a hemocytometer.

### 2.8. Cell Labelling and Co-Culture Experiments

To label cells with CellTrace Far Red dye (ThermoFisher Scientific), fibroblasts were incubated in Hanks’ Balanced Saline Solution (HBSS) containing 2 μM Cell Trace dye at 37 °C in air containing 5% CO_2_ for 20 min. The cells were then washed twice with DMEM containing 10% *v*/*v* FCS to quench any free dye in solution, before being replenished in DMEM containing 0.4% *v*/*v* FCS for an additional 24 h. Cells were then senescence-induced before being harvested by trypsinization and reseeded with untreated fibroblasts into 8-chamber slides. Co-cultures were maintained in the same DMEM containing 0.4% *v*/*v* FCS for 1, 5 or 7 d.

### 2.9. Protein Immunoblotting

Fibroblast cultures were lysed with RIPA lysis buffer containing protease (Roche, Basel, Switzerland) and phosphatase (Sigma-Aldrich) inhibitors and centrifuged at 14,000× *g* for 10 min at 4 °C to remove cellular debris. Protein concentrations were measured using the Pierce BCA protein assay kit (Thermo Scientific). Equal amounts of protein were loaded with 4× Laemmli Sample Buffer (Bio-Rad) and subjected to SDS polyacrylamide gel electrophoresis (SDS-PAGE) using 4–15% Mini-Protean TGX 10- or 15-well gels (BioRad). Protein was transferred to a nitrocellulose membrane (BioRad) in a semi-dry transfer unit (Hoefer™). Membranes were cut horizontally into three sections according to where ColIα1 (top), β-actin (middle) and p21 (bottom) migrate, before the membranes were blocked in a 5% *w*/*v* BSA/ 2.5% *w*/*v* non-fat milk powder in Tris-buffered saline and 0.5% *v*/*v* Tween 20 (TBST) buffer for 1 h. Membranes were then separately incubated overnight at 4 °C with anti-p21 (Cell Signaling Technology #2946, diluted 1 in 1000), ColIα1 (Cell Signaling Technology #84336, diluted 1 in 1000) or β-actin (Abcam #8227, diluted 1:10,000) immunoglobulin (IgG). Membranes were then washed with TBST before incubation with IgG secondary antibodies conjugated to horseradish peroxidase (HRP) for 1 h at room temperature. After additional TBST washes, membranes were imaged using SuperSignal West Femto Maximum Sensitivity Substrate reagents (ThermoFisher Scientific) on a ChemiDoc MP Imaging System (BioRad). The intensity of bands of interest were quantified using Image Lab version 5.1 (BioRad) and normalised to β-actin for densitometric analysis.

### 2.10. Immunofluorescence Staining

Fibroblasts were fixed with 4% paraformaldehyde for 15 min and washed in 50 mM glycine/PBS before being permeabilised in 0.5% *v*/*v* Triton X-100 in PBS for 10 min. Non-specific antibody binding was blocked by addition of 10% *v*/*v* goat serum in PBS for 1 h at room temperature. Cells were then incubated with primary antibodies for either p21 (Cell Signalling Technology #2947, rabbit, diluted 1 in 800) or H2AXγ (Cell Signalling Technology #80312, mouse, diluted 1 in 100) at 4 °C overnight followed by incubation with the appropriate Alexa Fluor 488 or 555 anti-rabbit or -mouse IgG conjugates (Cell Signalling Technology) for 1 h at room temperature. ProLong Gold mountant (Thermo Scientific) with DAPI nuclear stain was used to fix cells. Images were visualised using an Axio Imager 2 (Zeiss, Oberkochen, Baden-Württemberg, Germany). Quantitation was achieved using Fiji software (NIH) macros with specific plugins to specifically measure the percentage area of nucleus associated with fluorescence from either p21 or H2AXγ. Nuclei from cells labelled with CellTrace Far Red dye were excluded from quantitation.

### 2.11. ELISA

Levels of IL-6 in CM were measured by specific sandwich enzyme-linked immunosorbent assays (ELISA) using commercial kits (RnDSystems, Minneapolis, MN, USA) according to the manufacturer’s instructions.

### 2.12. PCR Analysis

Levels of mRNA were analysed by real time polymerase chain reaction (PCR) after purification from cells using RNeasy mini spin columns (Qiagen, Hilden, North Rhine-Westphalia, Germany) and reverse transcription into cDNA using the iScript Advanced cDNA kit (BioRad, Hercules, CA, USA). DNA was amplified by qPCR using the iTaq Universal SYBR Green Supermix (BioRad) in a QuantStudio 6 Pro real time PCR system (ThermoFisher) with the relevant PCR primers (sequences provided in Schuliga et al. [15,18]). For RNA quantitation, the threshold cycle (CT) value determined for each gene of each sample was normalised against that obtained for 18S rRNA, used as an internal control. The level of mRNA for a particular gene is proportional to 2^−(^^ΔCT)^, where ΔCT is the difference between the CT values of the target gene and 18S rRNA.

### 2.13. Statistical Analysis

Grouped data are presented as scatter dot plots with the horizontal bar designating the median. With the exception of experiments using A549 cells, *n* represented individual experiments conducted using cells from separate IPF patients or control donors. For A549 cells, two separate experiments were conducted with three biological replicates per treatment group per experiment. For data sets equal to or greater than *n* = 5, comparisons between two groups were analysed by the non-parametric Wilcoxon matched pairs signed rank or Mann–Whitney U tests (Graphpad Prism 5.0, Graphpad, San Diego, CA, USA) as appropriate. A value of *p* < 0.05 was considered to be statistically significant.

## 3. Results

### 3.1. Senescent Fibroblasts Accumulate in Areas of Collagen Deposition in IPF Lung

**Figure 1:**
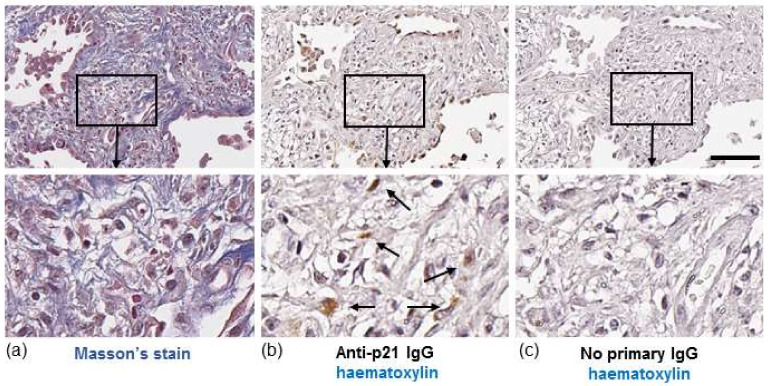
Histochemical staining of IPF lung. (**a**, **left panel**) Masson’s trichrome staining for collagen in a lung section from an IPF patient. Images show fibrotic mass comprising collagen and fibroblasts. (**b**, **middle panel**) A serial section from the same region of lung stained with hematoxylin (blue/purple) and p21 (brown). Images show p21-positive fibroblast-like cells (brown, highlighted by arrows). (**c**, **right panel**) No primary antibody control. Scale bar = 100 μm. Images in bottom row are enlarged areas of corresponding images in top row (10× magnification).

**Figure 2:**
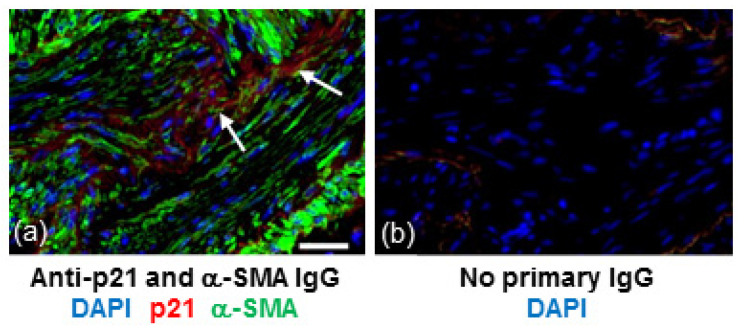
p21 is detected in areas of α-SMA positive cells within fibrotic regions of IPF lung. (**a**) Section of lung tissue from a patient with IPF immunostained for p21 (red) and α-SMA (green) and counterstained with DAPI (blue). Arrows show p21 immunoreactivity within clusters of α-SMA positive cells. (**b**) No IgG control. Scale bar = 25 μm.

In IPF, both fibroblasts and collagen accumulate predominantly in the lung parenchyma. To identify whether senescent fibroblasts are present in areas of collagen deposition we stained serial lung sections from an IPF patient for collagen or the senescent marker, p21. Histological Masson’s trichrome (blue) staining highlighted gross collagen deposition in the IPF lung (Figure 1a). DAB staining of a corresponding serial section with hematoxylin counterstaining revealed p21-positive fibroblast-like cells with elongated irregular nuclei residing within the collagenous region of the lung (Figure 1b). Similar patterns of p21 were detected in the lung of two other IPF patients examined (images not shown). Immunofluorescence detection of p21 in serial sections of lung tissue from an IPF patient shows localisation of p21 within cells that are positive for α-smooth muscle actin (α-SMA), a marker of myofibroblasts (Figure 2).

### 3.2. Non-Senescent Fibroblasts Cultured with Conditioned Medium from H_2_O_2_-Treated Lung Fibroblasts Have Increased Levels of Senescence Markers

**Figure 3:**
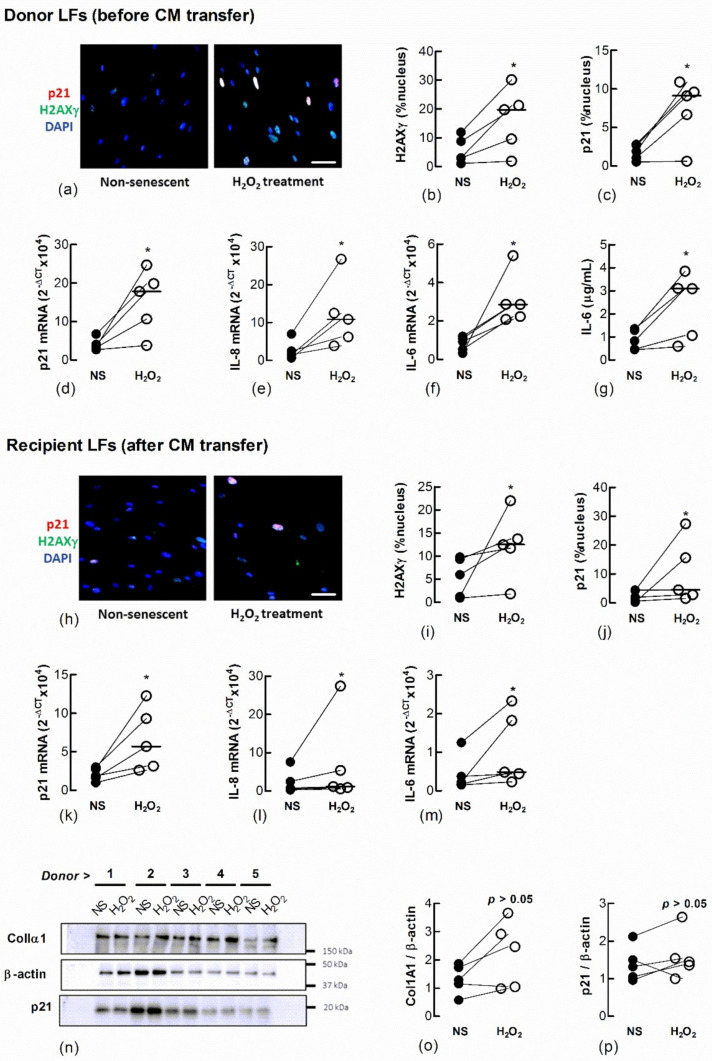
Conditioned medium from H_2_O_2_-treated fibroblasts increases markers of senescence in naïve fibroblasts. Sub-confluent naïve recipient Ctrl-LFs cultured with CM from untreated fibroblasts non-senescent (NS) or senescence-induced donor fibroblasts (H_2_O_2_, 150 μM) for 3 d. (**a**–**c**) Representative immunofluorescence images from an individual donor culture (blue = DAPI, red = p21 and green = H2AXγ) and grouped quantitative data measuring nuclear localisation of H2AXγ and p21 in donor Ctrl-LFs before CM transfer. (**d**–**g**) Corresponding levels of p21, IL-6 and IL-8 mRNA and IL-6 protein in donor fibroblasts. (**h**–**m**) Senescence markers in naïve recipient Ctrl-LFs 3 d cultured with CM. (**n**) Western blots showing ColIα1, p21 and the loading control, β-actin in lysates from naïve Ctrl-LFs treated with CM. (**o**,**p**) Densitometry of ColIα1 or p21 after normalisation to β-actin for individual donors. (* *p* < 0.05). Scale bar = 50 μm.

To determine if senescent LFs secrete factors that induce senescence in naïve fibroblasts we induced senescence by treating Ctrl-LFs with H_2_O_2_ and examined the effect of CM from these cells on naïve fibroblasts (Figure 3). They expressed a senescent phenotype 3 days after a 2 h exposure to 150 μM H_2_O_2_. The cells had an increase in the expression and nuclear localisation of p21, formation of nuclear H2AXγ and the expression or production of IL-6 and IL-8, archetypal SASP cytokines (Figure 3a–g). When naïve Ctrl-LFs were cultured with CM from H_2_O_2_-treated Ctrl-LFs for an additional 3 days, the cells showed increases in senescence markers, including levels of p21, IL-6 and IL-8 mRNA and increases in the levels and nuclear localisation of H2AXγ and p21 (Figure 3h–m) (*p* < 0.05, *n* = 5). The levels of p21 and ColIα1 protein in cell lysates were also higher in four of five cultures compared to CM from non-treated cells (Figure 3n–p).

### 3.3. Ctrl-LFs Become Senescent after Exposure to Conditioned Medium from IPF-LFs

**Figure 4:**
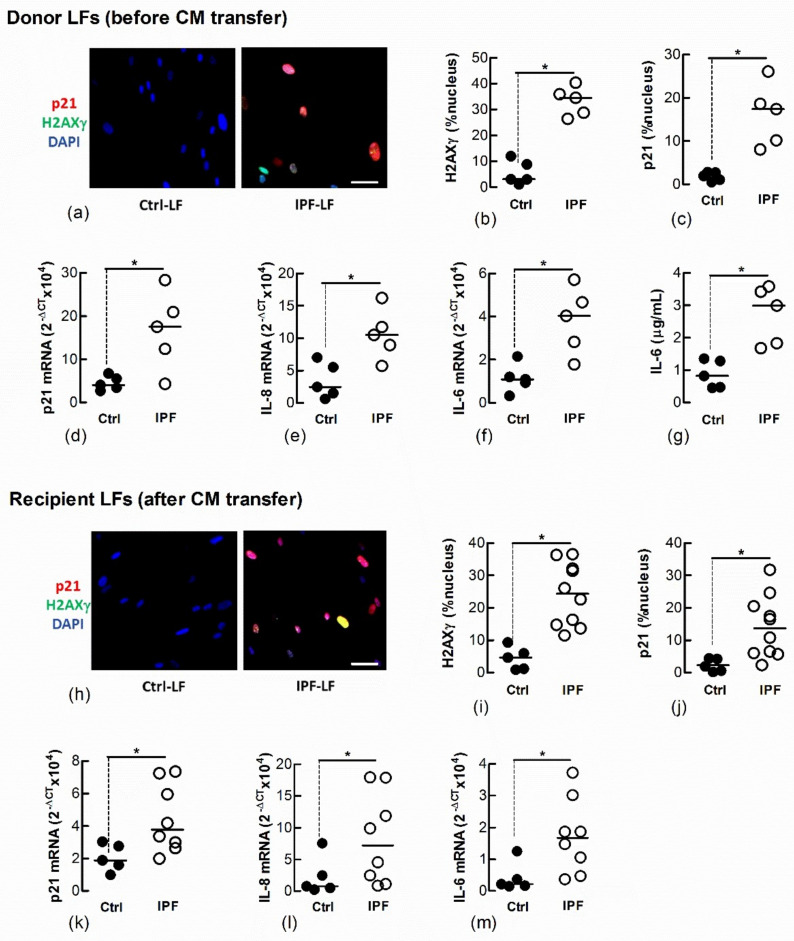
Conditioned medium from IPF-LFs induces senescence in naïve Ctrl-LFs. Sub-confluent naïve recipient Ctrl-LFs were exposed to CM from Ctrl- or IPF-LFs for 3 d (*n* = 5–10). (**a**–**g**) Levels of senescence markers in donor Ctrl- and IPF-LFs prior to culture with CM. Representative immunofluorescence images from Ctrl- and IPF-LF cultures show nuclear localisation of H2AXγ and p21 (blue = DAPI, red = p21 and green = H2AXγ). (**h**–**m**) Levels of senescence markers in naïve Ctrl-LFs 3 d after culture with CM. Each data point for the IPF treatment group represents a unique combination of CM from a “donor” IPF patient and recipient Ctrl-LF. CM from LFs of five separate IPF patients were used. (* *p* < 0.05). Scale bar = 50 μm.

We and other laboratories have shown that IPF-LFs have a higher baseline of senescence than age-matched Ctrl-LFs [12,14]. To further explore the transfer of LF senescence, naïve Ctrl-LFs were cultured with CM from IPF-LFs for 3 days. The IPF-LFs used in this study were derived from five separate IPF patients and exhibit a senescence-like phenotype, as shown by the increased expression and/or levels of p21, nuclear H2AXγ, IL-6 and IL-8 when compared with Ctrl-LFs (*n* = 5) (Figure 4a–g). CM from IPF-LFs induced senescence in naïve recipient Ctrl-LFs (Figure 4h–m).

### 3.4. Naïve Fibroblasts Become Senescent When Exposed to Conditioned Medium from Etoposide-Treated Fibroblasts or AECs

**Figure 5:**
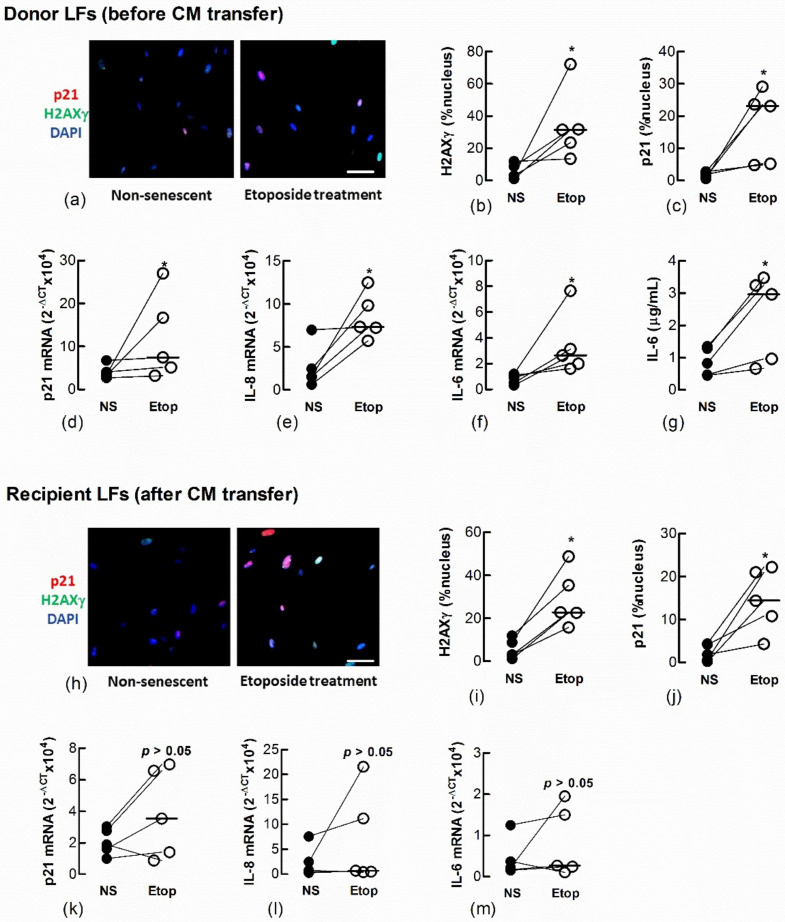
Conditioned medium from etoposide-treated fibroblasts induces senescence in naïve fibroblasts. Sub-confluent naïve recipient Ctrl-LFs were exposed to CM from untreated, non-senescent fibroblasts (NS) or senescence-induced fibroblasts (Etoposide, 10 μM). (**a**–**g**) Levels and/or expression of the senescence markers, p21, nuclear H2AXγ, IL-6 and IL-8 in Ctrl-LFs 3 d after etoposide treatment. Representative immunofluorescence images from an individual donor Ctrl-LF culture showing staining for H2AXγ and p21 (blue = DAPI, red = p21 and green = H2AXγ). (**h**–**m**) Levels or expression of senescence markers in naïve recipient Ctrl-LFs 3 d after culture in CM. (* *p* < 0.05). Scale bar = 50 μm.

**Figure 6:**
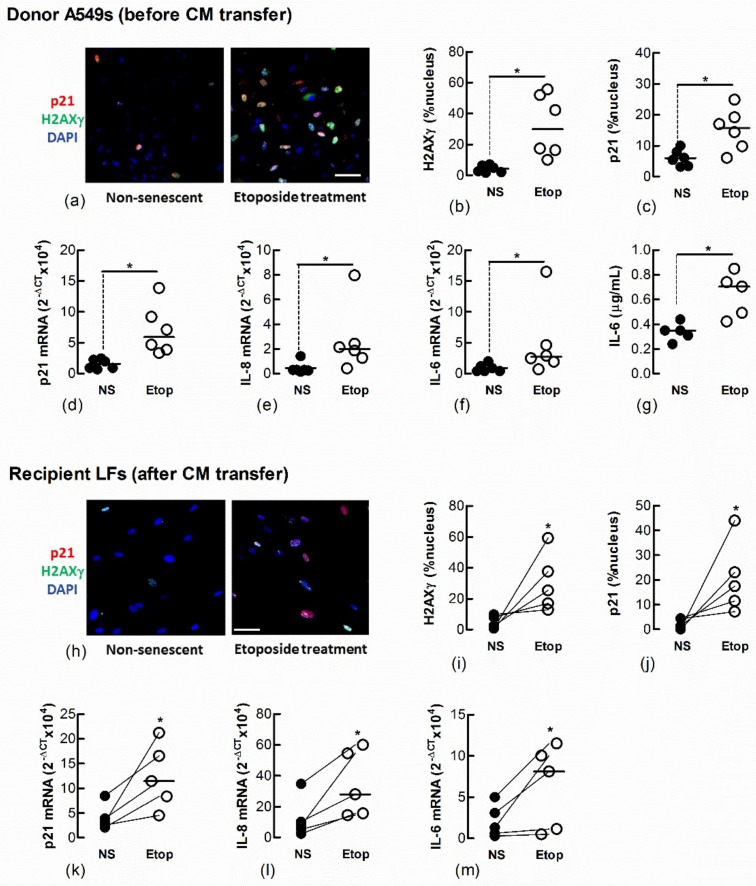
Conditioned medium from etoposide-treated A549 cells induces markers of senescence in naïve fibroblasts. Sub-confluent naïve recipient Ctrl-LFs were exposed to CM from untreated, non-senescent (NS) A549 cells or senescence-induced A549 cells (Etoposide, 10 μM). (**a**–**g**) Levels or expression of the senescence markers, p21, nuclear H2AXγ, IL-8 and IL-6 in A549 donor cells 3 d after etoposide treatment. Representative immunofluorescence images show the nuclear localisation of H2AXγ and p21 (blue = DAPI, red = p21 and green = H2AXγ). (**h**–**m**) Expression and levels of senescence markers in naïve recipient Ctrl-LFs 3 d after culture with A549-derived CM. (* *p* < 0.05). Scale bar = 50 μm.

We also showed a transfer of senescence to naïve recipient Ctrl-LFs by treating Ctrl-LFs with CM from either LFs (Figure 5a–g) or AECs (Figure 6a–g) treated with the DNA topisomerase inhibitor, etoposide (Etop); an inducer of DNA damage and senescence (Figure 5h–m and Figure 6h–m) [14].

### 3.5. Condition Medium from Senescent Fibroblasts Attenuates the Proliferation of Naïve Fibroblasts

**Figure 7:**
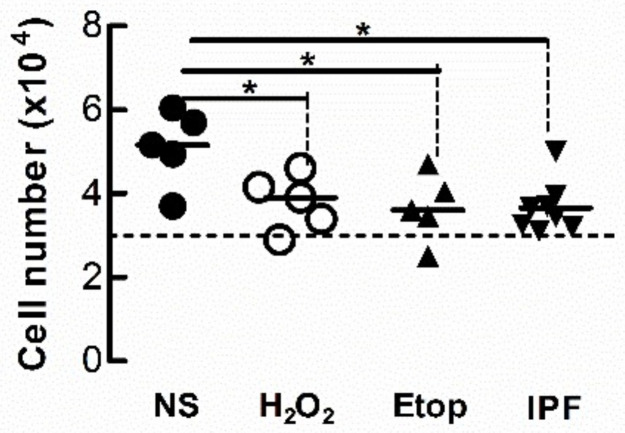
Conditioned medium from senescent fibroblasts reduces proliferation of naïve fibroblasts. Sub-confluent naïve Ctrl-LFs were exposed to CM (containing 0.4% FCS) from senescence-induced Ctrl-LFs using H_2_O_2_ or etoposide, or IPF-LFs with a higher baseline of senescence for 3 d before the medium was replenished with fresh medium containing 5% FCS. After an additional 2 days, cells were harvested and enumerated. The dashed horizontal line represents the initial number of cells seeded per well. (* *p* < 0.05).

Cell-cycle arrest is a defining feature of cellular senescence. We explored whether CM from senescent LFs diminishes the proliferative response of naïve fibroblasts. After 3 days exposure to CM from senescent donor fibroblasts, recipient Ctrl-LFs were grown in fresh medium containing 5% FCS for an additional 48 h then counted. The number of Ctrl-LFs exposed to CM from donor LFs treated with either etoposide or H_2_O_2_ were lower than LFs exposed to CM from untreated Ctrl-LFs (Figure 7). The number of Ctrl-LFs cultured with CM from senescent-prone donor IPF-LFs were also lower than LFs exposed to CM from Ctrl-LFs (Figure 7).

### 3.6. Naïve Lung Fibroblasts Become Senescent When Co-Cultured with H_2_O_2_-Treated Fibroblasts

**Figure 8:**
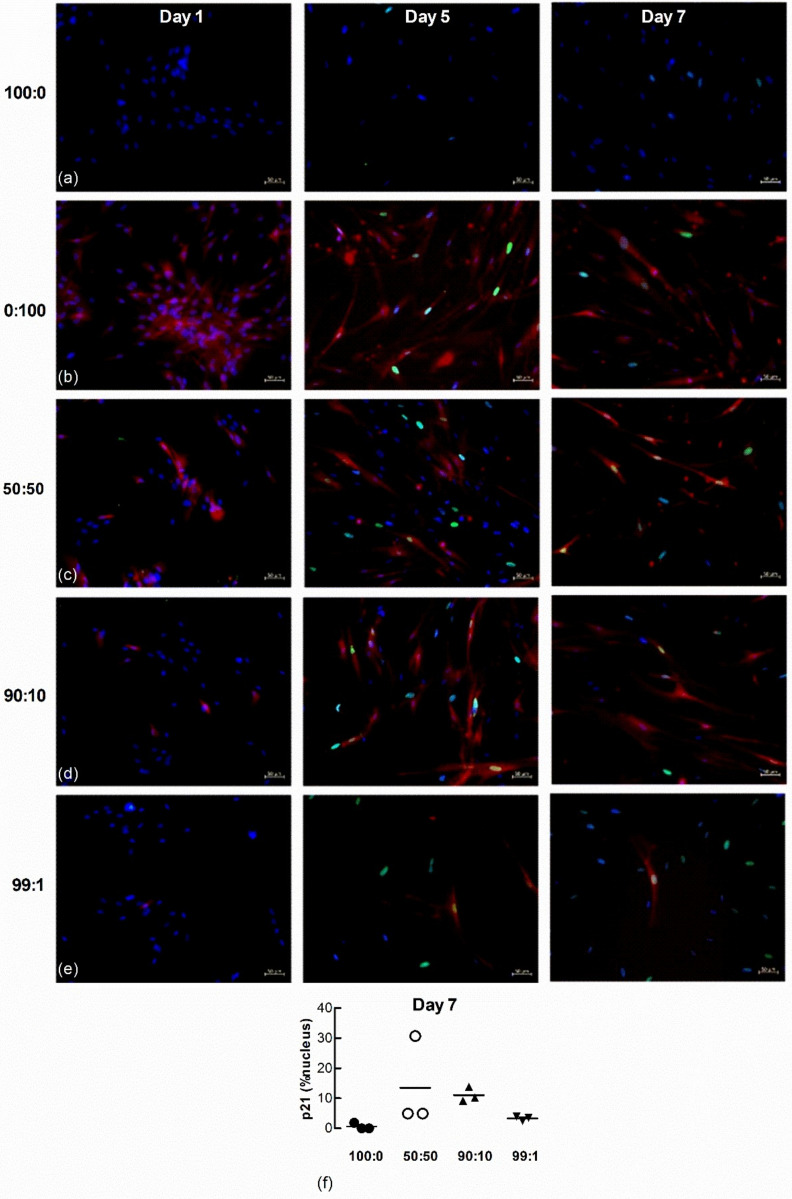
Nuclear p21 accumulates in naïve fibroblasts co-cultured with senescence-induced fibroblasts. Untreated and CellTrace Far Red dye loaded senescence-induced fibroblasts were seeded together at different ratios and maintained in culture for 1, 5 and 7 d (blue = DAPI, green = p21, red = senescence-induced fibroblasts). (**a**) Monocultures of untreated fibroblasts (100:0). (**b**) Monocultures of senescence-induced fibroblasts (0:100). (**c**–**e**) Co-cultures established from different seeding ratios of untreated and senescence-induced cells (50:50, 90:10 and 99:1). Scale bar = 50 µm. (**f**) The percentage area of nucleus associated with p21 in untreated naive fibroblasts after co-culture with senescence-induced fibroblasts for 7 d (*n* = 3).

To further examine senescence-induced senescence of lung fibroblasts, we co-cultured naïve non-senescent cells with senescence-induced cells loaded with the CellTrace Far Red dye. The accumulation of nuclear p21 and H2AXγ DNA damage foci in naïve non-senescent fibroblasts after co-culture with senescence-induced fibroblasts were examined by immunofluorescence microscopy 1, 5 and 7 d after seeding at the following ratios of non-senescent to senescence-induced cells: (a) 100 to 0; (b) 0 to 100; (c) 50 to 50; (d) 90 to 10; and (e) 99 to 1. There was no p21 (green) staining within nuclei (blue) in both untreated and H_2_O_2_-treated (red) populations 1 d after seeding (Figure 8a–e). Naïve non-treated cells co-cultured with increasing ratios of H_2_0_2_-treated cells demonstrated increasing levels of p21 after 5 and 7 days. Seeding density appeared to influence the degree of senescence-induced senescence as the quantifiable accumulation of nuclear p21 in naïve non-senescent cells was increased in co-cultures comprised of higher ratios of H_2_O_2_-treated fibroblasts (50:50 and 90:10) than lower ratios (99:1) (Figure 8f, *n* = 3).

**Figure 9:**
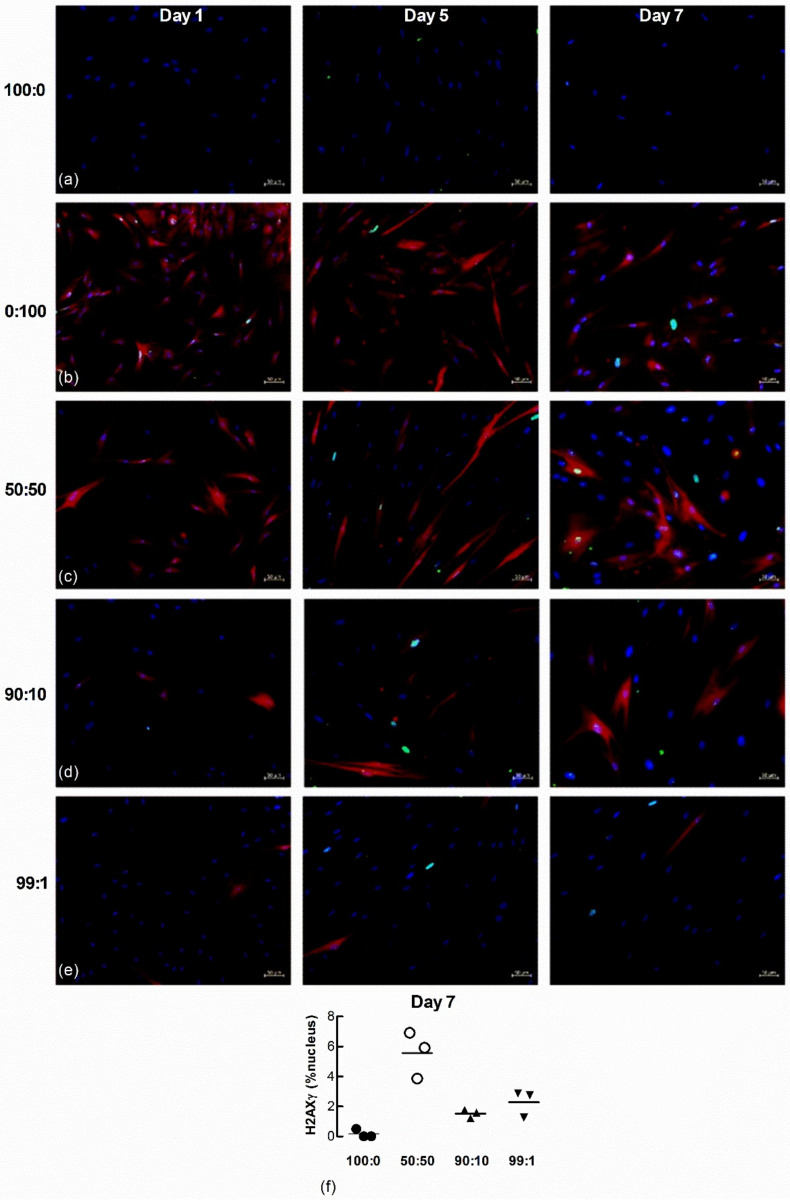
Nuclear H2AXγ increases in untreated naïve fibroblasts co-cultured with senescence-induced fibroblasts. Untreated and fluorescent-labelled senescence-induced fibroblasts were co-cultured at different ratios and maintained in culture for 1, 5 and 7 d (blue = DAPI, green = H2AXγ, red = senescence-induced fibroblasts). (**a**,**b**) Monocultures of untreated (100:0) and senescence-induced fibroblasts (0:100). (**c**–**e**) Co-cultures of untreated and senescence-induced fibroblasts at different ratios (50:50, 90:10 and 99:1). Scale bar = 50 µm. (**f**) The percentage area of nucleus associated with H2AXγ in non-treated naïve fibroblasts after co-culture with senescence-induced fibroblasts for 7 d (*n* = 3).

Nuclear H2AXγ, another marker of senescence, was also examined in the co-cultured cells. A larger number of H_2_0_2_-treated fibroblasts expressed nuclear H2AXγ staining 1 d after seeding (Figure 9a–e) but by 7 days a larger proportion of untreated LFs co-cultured with H_2_O_2_-treated cells expressed H2AXγ, even at the highest ratio of non-treated to senescence-induced cells seeded (99:1) (Figure 9f, *n* = 3).

## 4. Discussion

Fibroblast senescence is increasingly recognised as a feature of IPF, but the origin and role of senescence in IPF pathogenesis is unclear [19]. From our observation of senescent fibroblast-like cells in areas of dense collagen in IPF-lung, we hypothesised that senescent cells accumulate in the lung due the phenomenon of senescence-induced senescence. In this study we demonstrated that senescent lung fibroblasts and AECs induced key features of senescence in normal lung fibroblasts including increased DNA damage and expression of p21 and SASP cytokines, as well as reduced cell proliferation.

Senescent fibroblasts had previously been identified in histological sections of IPF lung [9,10]. In this study we used immunostaining for p21, a cyclin-dependent kinase inhibitor that promotes cell-cycle arrest, to show that senescent cells are situated in clusters of α-SMA positive cells within dense fibrotic areas of IPF lung. The α-SMA positive cells are likely to be myofibroblasts, activated fibroblasts that produce collagen [20]. These observations highlighted that senescent cells accumulate in collagenous areas, suggesting the occurrence of senescence-induced senescence in vivo and an association between fibroblast senescence and collagen deposition.

To determine if senescent LFs could accumulate through senescence-induced senescence in IPF lung, we cultured human adult lung fibroblasts with CM from senescent LFs, A549 AECs or IPF cells and also co-cultured senescent cells with normal LFs. CM from H_2_O_2_-induced senescent cells induced features of senescence in LFs including increased nuclear localisation of H2AXγ and p21, SASP cytokine (IL-6 and IL-8) and p21 expression, as well as inhibited cell proliferation. The levels of p21 and ColIα1 protein were also increased in four out of the five control LF cell lysates. Indeed, the CM of IPF-LFs from five separate patients examined also induced a senescence response in recipient naïve Ctrl-LFs as assessed using the aforementioned indices of senescence (except protein levels of p21 and ColIα1, which were not measured). This transfer of senescence was anticipated as IPF-LFs had previously been reported to have a higher baseline of senescence when compared to Ctrl-LFs, as was confirmed in this study [12,14]. The universality of the senescence-induced senescence phenomenon involving LFs was shown using CM from etoposide-treated lung fibroblasts or A549 AECs. The observation that senescent AECs induce senescence in LFs is particularly interesting as lung epithelial cell senescence may drive the aberrant wound healing response that leads to fibrosis in IPF [6,7,19,20]. Recently, populations of KRT17+ epithelial cells expressing markers of senescence have been identified in the lungs of IPF patients as an intermediate phenotype between type 2 and 1 AECs [21,22]. These progenitor cells are possibly “stuck” in a transitional state, which prevents them from undergoing differentiation and playing a role in normal tissue repair [23]. Instead, the senescent KRT17+ lung epithelial cells may contribute to the formation of a fibrotic niche through production of SASP mediators that can influence neighboring lung fibroblasts, including their transition into a senescence phenotype. Which bioactive molecules mediate the transfer of senescence from AECs and LFs to naïve LFs remains to be elucidated, but a diverse range of factors of the SASP is likely to be involved, including ROS, cytokines and growth factors such as IL-6 and TGF-β and associated molecular patterns (DAMPs) i.e., damaged mitochondrial DNA fragments and ECM peptides [5,14,15,24].

The transfer of senescence to LFs was also explored in this study using co-cultures comprised of different ratios of H_2_O_2_-induced senescent and non-senescent cells. Although the sample size was small (*n* = 3), p21 nuclear staining analysis suggested that more senescence was induced in those cultures with a higher ratio of H_2_O_2_-treated senescent cells. However, the observation that even a small proportion of senescent cells (i.e., 1% of the total cell population) can lead to a substantial increase in overall nuclear p21 accumulation in the untreated cell population perhaps best illustrates the phenomenon of senescence-induced senescence. Interestingly, the transfer of senescence appears evenly distributed amongst the untreated cell population, not accumulating in clusters of cells. These observations along with data from the CM culture experiments provide evidence that bioactive molecules secreted from senescent cells drive the transfer of senescence; a process that does not require cell to cell contact.

A time course for H2AXγ staining revealed cumulative DNA damage in untreated cells when co-cultured with senescence-induced cells. H2AXγ foci were readily seen in the H_2_0_2_-treated cells 24 h after seeding, whereas H2AXγ was undetectable in the untreated cells. However, H2AXγ staining increased over time in untreated cells co-cultured with H_2_O_2_-treated cells, suggesting cumulative DNA damage.

We cannot confirm that senescence was induced in untreated cells in co-culture as only two markers of senescence, p21 and H2AXγ were assessed. Although increased levels of p21 and cell-cycle arrest are core features of senescence more markers need to be examined to confirm senescence. DNA damage, as indicated by the presence of nuclear H2AXγ is likely to initiate the DNA damage response (DDR) that leads to increases in p21 in senescence. However, H2AXγ can also increase in apoptosis, albeit its distribution in this cellular process is diffuse, not punctate as it is in senescence [24]. The emergence of H2AXγ foci before p21 in the nucleus of non-treated cells in co-culture suggests a pre-senescence state; most likely caused by alterations in the secretome of H_2_O_2_-induced donor cells [16].

The oxidant (H_2_O_2_)-induced senescent fibroblast model used in this study resulted in increased ROS generation, which had previously been shown to be exchanged through cytoplasmic bridges to induce senescence in recipient cells [5]. It should be noted that both etoposide-treated lung fibroblasts and lung fibroblasts derived from IPF patients not only exhibited increased senescence but also mitochondrial stress and superoxide production [14]. Indeed, studies with lung fibroblasts and epithelial cells showed a cyclical, mutually reinforcing relationship between senescence and mitochondrial dysfunction; a viscous circle involving increased production of mitochondrial-derived ROS and activation of the mammalian target of rapamycin complex 1 (mTORC1) [14,16,25]. The latter is downstream of the DDR and regulates mitochondrial homeostasis in an auto-amplifying loop that perpetuates senescence [26]. There has been a long standing acknowledgment of the role of oxidative stress and mitochondrial dysfunction in fibrosis and ageing and attenuated redox regulation is well documented in the context of IPF [7,9,27]. Future experiments should confirm senescence induction in the untreated population and use ROS scavengers during co-culture experiments to determine if ROS is responsible for inducing senescence.

Our findings support other published data that senescent cells can induce senescence in neighboring cells in vitro. Previous studies used IMR90 fibroblasts [25], MRC5 fibroblasts [5] and animal models [27,28]. However, this study showed for the first time senescence-induced senescence using primary cultures of lung fibroblasts from IPF patients as well as age appropriate human adult control donors. These data suggest that the extent of senescence within an IPF lung may not only be the result of an injurious event (i.e., from inhaled pollutants) but may also have been propagated from senescent to non-senescent cells as the disease progresses; and that this transfer of senescence may be mediated by secreted bioactive mediators of the SASP.

## Figures and Tables

**Table 1:** Characteristics of fibroblast samples.

Fibroblast Sample No.	Sample Type	Age	Sex	Diagnosis	Smoking History (Pack yrs)
IPF patient 1	Tissue section	65	M	IPF	Ex (40)
IPF patient 2	Tissue section and fibroblast culture	63	M	IPF	Ex (20)
IPF patient 3	Tissue section	57	M	IPF	Ex (48)
IPF patient 4	Tissue section	70	M	IPF	N/A
IPF patient 5	Fibroblast culture	61	M	IPF	Ex (2)
IPF patient 6	Fibroblast culture	67	M	IPF	Never
IPF patient 7	Fibroblast culture	70	F	IPF	Ex (41)
IPF patient 8	Fibroblast culture	64	F	IPF	Never
Donor 1	Fibroblast culture	39	N/A	Ctrl	Ex (15)
Donor 2	Fibroblast culture	66	F	Ctrl	Ex (28)
Donor 3	Fibroblast culture	35	N/A	Ctrl	N/A
Donor 4	Fibroblast culture	67	F	Ctrl	N/A
Donor 5	Fibroblast culture	61	F	Ctrl	N/A
Donor 6	Fibroblast culture	63	M	Ctrl	Ex

Characteristics include age, gender, diagnosis and smoking history (Pack years), Ctrl = control, N/A = data not available.

## Data Availability

All data generated or analyzed during this study are included in this published article.
